# Molecular Identification and Evaluation of the Genetic Diversity of *Dendrobium* Species Collected in Southern Vietnam

**DOI:** 10.3390/biology9040076

**Published:** 2020-04-10

**Authors:** Nhu-Hoa Nguyen, Huyen-Trang Vu, Ngoc-Diep Le, Thanh-Diem Nguyen, Hoa-Xo Duong, Hoang-Dung Tran

**Affiliations:** 1 Faculty of Biology, Ho Chi Minh City University of Education, 280 An Duong Vuong Street, Ward 4, District 5, Ho Chi Minh 72711, Vietnam; 2 Faculty of Biotechnology, Nguyen Tat Thanh University, 298A-300A Nguyen Tat Thanh Street, Ward 13, District 4, Ho Chi Minh 72820, Vietnam; vthtrang@ntt.edu.vn (H.-T.V.); lethingocdiep2807@gmail.com (N.-D.L.); nthanhdiem1708@gmail.com (T.-D.N.); 3 Biotechnology Center of Ho Chi Minh City, 2374 Highway 1, Quarter 2, Ward Trung My Tay, District 12, Ho Chi Minh 71507, Vietnam; hoaxo912@gmail.com

**Keywords:** *Dendrobium*, ITS, ITS2, *mat*K, *rbc*L, *trn*H-*psb*A, southern Vietnam, molecular identification, genetic diversity, DNA barcoding

## Abstract

*Dendrobium* has been widely used not only as ornamental plants but also as food and medicines. The identification and evaluation of the genetic diversity of *Dendrobium* species support the conservation of genetic resources of endemic *Dendrobium* species. Uniquely identifying *Dendrobium* species used as medicines helps avoid misuse of medicinal herbs. However, it is challenging to identify *Dendrobium* species morphologically during their immature stage. Based on the DNA barcoding method, it is now possible to efficiently identify species in a shorter time. In this study, the genetic diversity of 76 *Dendrobium* samples from Southern Vietnam was investigated based on the ITS (Internal transcribed spacer), ITS2, *mat*K (Maturase_K), *rbc*L (ribulose-bisphosphate carboxylase large subunit) and *trn*H-*psb*A (the internal space of the gene coding histidine transfer RNA (trnH) and gene coding protein D1, a polypeptide of the photosystem I reaction center (psaB)) regions. The ITS region was found to have the best identification potential. Nineteen out of 24 *Dendrobium* species were identified based on phylogenetic tree and Indel information of this region. Among these, seven identified species were used as medicinal herbs. The results of this research contributed to the conservation, propagation, and hybridization of indigenous *Dendrobium* species in Southern Vietnam.

## 1. Introduction

*Dendrobium* is among the most abundant genera of flowering plants with over 1148 known species, which ranks second in the orchid family, after the *Bulbophyllum* genus [1]. *Dendrobium* is diverse in shapes, colors, and sizes, and is hence considered as a favorite ornamental plant. Some *Dendrobium* species are also used as medicinal herbs, such as *D. densiflorum* and *D. chrysotoxum* [2]. Many studies on diverse *Dendrobium* species by geographic regions have been published for Australia [3,4], mainland Asia [5,6], China [7], Thailand [8,9], etc. These studies again confirm the rich diversity of the beautiful orchids.

The living environment of indigenous *Dendrobium* species in Vietnam is declining due to climate change and over-exploitation. An evaluation of genetic diversity and identification of *Dendrobium* species in Vietnam is critical for prompt conservation of this valuable genus. Morphology of *Dendrobium* species is similar at non-flowered stages, and hence misidentification often happens between conspecific species [10].

DNA barcoding is an effective method used in the identification of species, especially orchids. Many works have proved that the ITS region (Internal Transcribed Spacer) contains many genetic differences, so it is used to classify species and study relationships [10,11], particularly in *Dendrobium* [8,12]. The ITS2 region has been assessed as being able to clearly distinguish between *Dendrobium* species [13,14]. Two *mat*K and *rbc*L regions have also been identified as being able to identify species of the genus *Dendrobium* [4,10].

Tran (2015) conducted a diversity examination of indigenous *Dendrobium* species in Vietnam, mostly from Northern Vietnam, using ITS sequences [15]; 23 of 32 samples of *Dendrobium* were identified, among which four of nine unidentified samples were confirmed as *Dendrobium parishii* [15]. Nguyen et al. (2017) constructed a phylogenetic tree for the ITS region, and separated 12 samples of wild *Dendrobium* species collected in Southern Vietnam and 11 samples of imported *Dendrobium* from Thailand divided into two distinct groups. Those results corresponded to the classification by the traditional identification method [16]. Nguyen (2018) continued to evaluate ITS on the identification of 15 samples belonging to *Dendrobium thyrsiflorum,* which were delineated on single branches [17].

A large number of *Dendrobium* species in Southern Vietnam were evaluated for genetic diversity to improve conservation efforts in the current work. The identification capability of different sequences was also investigated. The results of our work contribute to the enrichment of the sequences in GenBank and have applications in practical conservation and management of genetic resources.

## 2. Materials and Methods

### 2.1. DNA Extraction and Amplification

The total DNA of 76 samples was isolated from fresh leaves by the Isolate II Plant DNA kit BIO-52069 (TBR Company, Ho Chi Minh City, Vietnam). Primers and thermocycling conditions used for the amplification of 4 regions, ITS, *mat*K, *rbc*L, *trn*H-*psb*A, are presented in Table 1. Components of the amplification reaction included 12.5 μL Taq DNA pol 2x-premix, 1 μL forward primer (5 μM–10 µM), 1 μL reverse primer (5 µM–10 μM), 1 μL DNA template and water to make 25 μL. PCR products were sequenced bi-directionally at Macrogen Company, Seoul, Korea.

### 2.2. Data Analysis

FinchTV software [22] was used to read and adjust nucleotide sequences. Forward and reverse sequences were combined into consensus sequences and aligned using Seaview 4.0 [23]. The ITS2 sequence was then extracted from the ITS sequence (Based on accession number JN388570.1) for analyses. The phylogenetic tree and variable parameters were calculated in MEGA 7.0 software [24] by using the Maximum Likelihood algorithm, following the 2-parameter Kimura model. The sequence of orchid species *Paphiopedilum delenatii* was used as an outgroup to root the tree. 

## 3. Results

### 3.1. Sample Collection, Amplification, and Sequencing

The 76 *Dendrobium* samples (Appendix A) were collected and divided into two groups: the collection of Biotechnology Center Ho Chi Minh (coded as TT) and the commercial samples (coded DT, PN). For ITS and *mat*K, all 76 collected samples were amplified. Since *rbc*L is a conserved region, only 35 samples from 30 species were amplified.

The PCR results in both ITS and *mat*K regions achieved success rates of 94.73% and 97.26%, respectively. Notably, the *rbc*L area had the best rate of 100%. Particularly in the *trn*H-*psb*A region, the PCR success rate was 82.19%. However, the amplification and sequencing of *trn*H-*psb*A were at low levels. Therefore, the data from the *trn*H-*psb*A region was not included in further analyses in the study.

### 3.2. Genetic Diversity Based on Nucleotide Polymorphism and Phylogenetic Analyses

Seventy-six samples of 30 collected *Dendrobium* species were included in the survey (Appendix A). For phylogenetic analysis, sequences of *Dendrobium* species from our study were compared with GenBank accessions (Accession numbers of GenBank sequences are shown in Appendix B). Based on the phylogenetic tree, individuals of the same species should cluster in the same branch that separates from the other species. In general, there was no conflict among the three constructed trees. However, the ITS gave the most separated branches. The ITS2 trees showed the same clusters as the ITS trees. Hence the ITS region was representatively analyzed for the divergence of *Dendrobium* species in Southern Vietnam.

On the ITS tree, samples of some species were grouped with their conspecific accessions from GenBank without mixing with other different species, i.e., *D. aloifolium, D. amabile, D. capillipes, D. chrysotoxum, D. crumenatum, D. crystallinum, D. densiflorum, D. farmeri, D. intricatum, D. parishii, D. secundum, D. sulcatum,* and *D. venustum*. *D. superbum* was the synonym name of *D. anosmum*. Hence their sequences were mixed up for both our samples and GenBank accessions and closely related to their sister *D. parishii*. As a result, the hybrid samples of *D. anosmum* × *parishii* and *D. anosmum* × *D. aphyllum* were also included in the phylogenetic branch of these species. *D. anosmum* × *parishii* is named *D. nestor,* and *D. anosmum* × *D. aphyllum* is named Adastra. The separation of *D. parishii* from *D. anosmum* was also reported by Tran et al. (2018) [15].

In both ITS and *mat*K phylogenetic trees, our sample of *D. salaccense* was not clustered with a group of the species accessions from GenBank. Interestingly, after searching other similar sequences from GenBank using the BLAST tool, our sample 24DT was homologous with *D. hancockii* at 99.71% in ITS data and 100% in *mat*K data (data not show). These two species have the same Vietnamese name, “Hoang Thao Truc”. Hence species confusion might happen during the sampling process. The scientific name of sample 24DTwas then corrected to *D. hancockii*.

**Figure 1:**
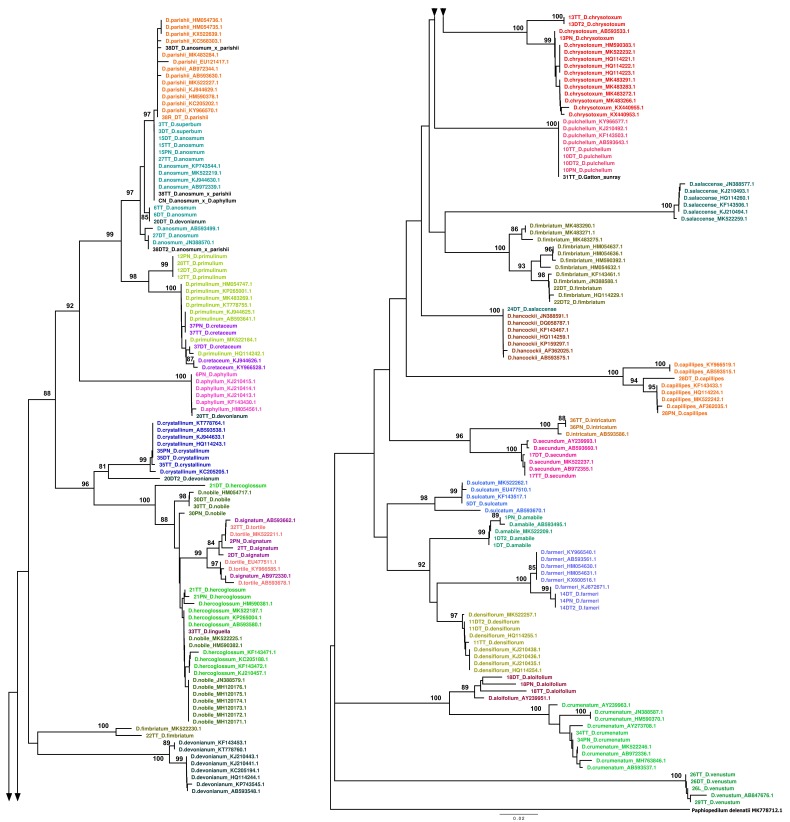
ITS tree is constructed base on the Maximum likelihood for *Dendrobium* collected at Southern Vietnam.

Among three samples of *D. fimbriatum*, two samples, 22DT and 22DT2, were grouped with other *D. fimbriatum* accessions from GenBank but sample 22TT was totally separated from this group. However, when compared to GenBank sequences, the remaining sample 22TT was also matched with another conspecific accession *D. fimbriatum* (MK522230.1) and was closely related to *D. devonianum* species (Figure 1). A further observation on the original alignment of these accessions showed that sequences of 22TT and *D. fimbriatum* (MK522230.1) were highly similar throughout the length and were fractionated into different regions, in which some fragments were similar to other *D. fimbriatum* accessions, some were similar to *D. devonianum* sequences, and some were distinct from all of others. This result proposed the conclusion that the 22TT sample was a hybrid of *D. fimbriatum* and *D. devonianum* as these two species share the same local habitat (Appendix A). Otherwise, *D. fimbriatum* might be diverted into different directions of the evolution process.

The variety *D. gatton sunray* was located in the same branch of *D. pulchellum* in both ITS and *matK* trees. *D. pulchellum* was crossed with *D. chrysotoxum* forming *D. illustre*. Then, *D. illustre* was crossed back with *D. pulchellum* to create *D. gatton sunray*. As a result, the hybrid, which contains lots of genetic characters from *D. pulchellum,* was grouped with its parent in phylogenetic trees.

Sequences of two species, *D. signatum* and *D. tortile,* were mixed up on the same branch. In terms of sexual morphology, their flowers are remarkably similar except that petals of species *D. tortile* are non-yellowed, more purple, and more twisted. Hence the molecular result was consistent with morphological features. *D. signatum* is sometimes called by the synonym scientific name *D. tortile* var. *hildebrandi* (Rolfe) T. Tang and F.T. Wang (1951). As a result, they had a very close genetic relationship. *D. hercoglossum* and *D. linguella,* are two synonym names of one species. On all phylogenetic trees, this species was closely related to *D. nobile*, *D. signatum,* and *D. tortile* and could not be completely distinguished.

Two species, *D. primulinum* and *D. cretaceum,* which have similar morphological features, were also close in genetic characters. The same situation also happened for two species, *D. primulinum* and *D. cretaceum*. The most divergent species was *D. devonianum* within our three conspecific samples, and even sequences of this species from GenBank were significantly separated into different branches on all ITS, *mat*K, and *rbc*L trees. Although there was not enough data to clarify this issue, the results suggested a hypothesis of breeding between *D. devonianum* and other species in nature. 

Briefly, there is a diversity of 28 species of *Dendrobium* in Southern Vietnam, including three hybrid species, which were investigated in this study. Among conspecific variations, there was also divergence, shown in different lengths of branches on the same cluster, i.e., species *D. amabile*, *D. secundum*, *D. capillipes*, *D. chrysotoxum,* and *D. crystallinum* (Figure 1).

### 3.3. Potential Sequences for Identification of Dendrobium Species in Southern Vietnam

Investigating genetic diversity of *Dendrobium* populations not only provides information for species management but also helps distinguish herbals and their adulterants, and significantly supports conservation by identifying and limiting trade of valuable and endangered species illegally. In this study, we assayed the potential of using sequences in species identification for practical conservation. In this analysis, 24 original species were included, except for three hybrids and the undetermined species *D. devonianum*. Twenty-three species were analyzed using *mat*K and *rbc*L data since *D. parishii* could not be amplified. The most critical measurement for evaluation was the species resolution of each region. Therefore, tree-based methods and indel information were combined to optimize achievement (Appendix C). Criteria such as variable sites, informative parsimony sites, and singleton sites were also recorded.

Both the ITS (56.65%) and ITS2 (52.89%) regions showed significantly high results in nucleotide polymorphism (variable sites) in comparison with *matK* (10.21%) and *rbc*L (6.58%) and *trn*H-*psb*A (8.31%) (Table 2). ITS2 was even more divergent than the full ITS region. This result was consistent with previous studies [25,26,27]. Based on the phylogenetic tree, the species identification by ITS2 (17 out of 24 species) was as effective as ITS (17 species).

**Figure 2:**
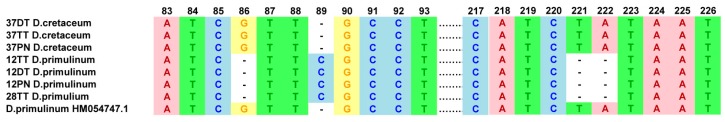
Insertion-deletion (indel) sites in sequences of *D. creatceum* and *D. primulinum* accessions.

From both ITS trees, three pairs of species were not separated, i.e., *D. cretaceum* and *D. primulinum*; *D. hercoglossum* and *D. nobile*; *D. tortile* and *D. signatum.* Our examination of insertion and deletion information from their full ITS sequences indicated the differences between *D. cretaceum* and *D. primulinum* at sites 86, 89, 221–222 (aligned with the complete ITS of *Dendrobium primulinum* HM054747.1) (shown in Figure 2), which did not exist in short version, ITS2. *D. primulinum* in this study had three deletions at sites 86, 221, 222, and 1 insertion at site 89. Therefore, these two species were distinguished, and ITS could identify 19 out of 24 species (79.16%). Although less divergent, the long ITS (15) contained more indel sites than the short ITS2 (12) and was proven to be useful in previous studies [28,29]. The combination of multiple loci as a single marker did not provide more species resolution. Finally, 19 out of 24 species were clearly identified, including *D. aloifolium, D. amabile, D. aphyllum, D. capillipes, D. chrysotoxum, D. cretaceum, D. crumenatum, D. crystallinum, D. densiflorum, D. farmeri, D. fimbriatum, D. intricatum, D. parishii, D. primulinum, D. pulchellum, D. hancockii, D. secundum, D. sulcatum,* and *D. venustum*.

In terms of best match/best close match methods in the evaluation of potential sequences for species identification, ITS2 gave the best results of the correct match, following by ITS and *mat*K. *rbc*L gave the lowest effect (Table 3).

The “best match/best close match” methods [30] are based on comparing the genetic distance of the analyzed sequences. The sequences that achieve intra-value are the smallest when compared to the order of the same species classified as correct. If this intra-value is also present when compared to other species, the sequence is classified as ambiguous. The sequences with intra-distances greater than inter-distances are categorized as incorrect. For the “best close match” method, a threshold value (%) is calculated based on all intra-distances, to determine the similarity of sequences. The sequences that do not meet this value (no match) will be deleted before being identified.

Both the *mat*K and *rbc*L regions are quite conserved sequence areas [31], and there was a similarity level higher than 97%, so when the threshold (3%) was set, no sequence was classified as “no match”. Meanwhile, the ITS and ITS2 sequences are sequences of high diversity, so the results (50 and 53, respectively) were higher than *mat*K and *rbc*L. When using the “best close match” with a threshold of 3% of the ITS2 region, the highest results were obtained (48 sequences), indicating that ITS2 was the most likely area of determination in the studied regions. Therefore, the ITS and ITS2 sequence regions were identified as potential barcodes.

In general, the results derived from best match/best close match methods (Appendix D) were consistent with branch forming of each sample on phylogenetic trees. For instance, on the tree (Figure 1), 30PN was separated in another branch from the group of 30DT and 30TT. The best match calculation from ITS data also reported sample 30PN *D. nobile* as incorrect while the two remain samples of that species, 30DT and 30TT, were correct. However, for this method, the relationship among species was not visualized as well as the tree-based method. For instance, we could not recognize that *D. anosmum* and *D. superbum* were clustered on the same branch as they are synonymous names of the same species, or *D. primulinum* with *D. cretaceum*. Hence, best match/best close match methods were used just for general evaluation of identification potential of a sequence.

## 4. Discussion

ITS was also used in previous studies on identification of *Dendrobium* species, among which some studies focused on medicinal species for distinguishing herbals and their adulterants [13,14]. In a previous study of Tran et al. (2018) [15], 19 out of 23 Vietnamese *Dendrobium* species (82.61%) were identified using the ITS marker (Appendix E). In our study, 28 species were considered in which 19 species (67.86%) were identified using the same marker ITS. Some species were identified in study of by Tran et al. (2018) but not in ours, i.e., *D. anosmum* and *D. nobile*. In contrast, two species, *D. amabile* and *D. fameri,* were clearly separated on monophyletic branches in our study but not in the previous research. Unidentified species were species with their sequences grouped with sequences of other species, forming paraphyletic or polyphyletic branches [28]. In the two studies, ITS could not resolve 100% of *Dendrobium* species. However it was the best in comparison with *mat*K and *rbc*L markers in our study. The difference of resolution effectiveness actually much depends on component of sample data. Sixteen species from our study were not included in study of Tran et al. (2018) and, vice versa, 11 species in their study were not in our collection. Tran and his colleagues collected samples from the whole of Vietnam and mostly from the northern areas, while our study collected species from southern regions. Besides, in the study of Tran et al. (2018), the sample size was small, with 32 specimens, and most of the sampled species (15 out of 23) were examined with only one representative sample. Therefore genetic diversity among conspecific individuals was not investigated in their study. In our study, 2 to 3 samples for each species, except for five species, *D. aphyllum*, *D. parishii*, *D. salaccense*, *D. sulcatum,* and *D. tortile*, were included for intra- and inter-specific genetic analyses. In short, our study results and the report of Tran et al. do not contradict each other but both gave a remarkable contribution to the sequence library of Vietnamese native *Dendrobium* diversity.

The intergenic spacer *trn*H-*psb*A was recommended by Yao et al. (2009) for the identification of 15 *Dendrobium* species [32] due to high divergence of sequences. In our study, this region was more difficult to amplify than other regions. The amplification rate was just 82.19% after repetition. This problem was consistent with the previous report of Gigot et al. (2007). *trn*H-*psb*A is supposed to contain too many tandem mononucleotide repeats which results in high levels of length variation and causes problem in amplification, bidirectional sequencing, and alignment [33].

The *mat*K and *rbc*L markers were used for this orchid group by Asahina et al. (2010) [10] and Moudi et al. (2013) [34]. Sigh et al. (2012) proposed the combination of three regions, *matK*, *rpo*B, and *rpo*C1 [35]. Among those barcoding regions, ITS was the most commonly used. [2,8,9,14,15,25,36,37,38,39]. Our results again confirmed the effect of ITS in the evaluation of genetic diversity and the identification of *Dendrobium* species not only in Southern Vietnam but also in other habitats.

## 5. Conclusions

The ITS2 region has the highest level of genetic diversity among the surveyed areas. In particular, the ITS region has more indels to help increase the ability to identify species. In general, both ITS and ITS2 have the most potential for assessment of genetic diversity and identification of *Dendrobium* species in Southern Vietnam. In this study, 19 *Dendrobium* species were recognized, many of which have high levels of diversity within the same species. Some species with easily confused morphological characteristics have also been redefined for accuracy based on molecular sequences. Research has contributed to increasing data in the library of *Dendrobium* of Vietnam and the world. Also, the two species with very similar morphologies can be distinguished, *D. primulinum* (used as medicinal herbs) and *D. creatceum,* to avoid confusion when using these species as medicinal herbs.

## Figures and Tables

**Table 1:** Primer sequences and the thermal cycles for amplification reactions of the ITS, *mat*K, *rbc*L, *trn*H-*psb*A regions.

Barcode	Primer Name	Primer Sequence	Thermal Cycle	Source
ITS	ITS1F	5′CTTGGTCATTTAGAGGAAGTAA3′	Denaturing: 94 °C/30 sec Annealing: 55 °C/40 sec Extending: 72 °C/1 min	[17,18]
	ITS4R	5′TCCTCCGCTTATTGATATGC3′		
*matK*	390F	5′CGATCTATTCATTCAATATTTC3′	Denaturing: 94 °C/1 min Annealing: 48 °C/30 sec Extending: 72 °C/1 min	[6,19]
	1326R	5′TCTAGCACACGAAAGTCGAAGT3′		
*rbcL*	aF	5′ATGTCACCACAAACAGAGACTAAAGC3′	Denaturing: 94 °C/30 sec Annealing: 55 °C/1 min Extending: 70 °C/1 min	[20]
	aR	5′CTTCTGCTACAAATAAGAATCGATCTCTCCA3′		
*trnH-psbA*	trnHF_05	5′CGCGCATGGTGGATTCACAATCC3′	Denaturing: 95 °C/30 sec Annealing: 5 °C/20 sec Extending: 72 °C/20 sec	[21]
	psbA3′f	5′GTTATGCATGAACGTAATGCTC3′		

**Table 2:** Comparison parameters of ITS (internal transcribed spacer), ITS2, *mat*K, *rbc*L, and *trn*H-*psb*A markers for identification of *Dendrobium* species.

Region	Length	Number of Samples	Number of Species	Variable Site (%)	Parsimony (%)	Single-ton (%)	Indel	Identified Species Based on the Phylogenetic Tree	Identified Species Based on the Phylogenetic Tree and Indel Information
ITS	639	68	24	362 (56.65)	338 (52.89)	24 (3.75)	15	17/24	19/24
ITS2	253	68	24	167 (66.00)	152 (60.07)	15 (5.92)	12	17/24	17/24
*matK*	822	65	23	84 (10.21)	53 (6.44)	31 (3.77)	3	12/23	12/23
*rbcL*	501	34	21	26 (6.58)	16 (4.59)	10 (1.99)	0	5/23	5/23
*trnH-psbA*	782	56	24	65 (8.31)	46 (5.88)	17 (2.17)	13	5/24	5/24

**Table 3:** The identification results of the “best match/ best close match” method.

Barcode	No Sequences	Best Match (%)			Best Close Match (%)			
		Correct	Ambiguous	Incorrect	Correct	Ambiguous	Incorrect	No Match
ITS	68	55 (80.88)	2 (2.94)	11 (16.17)	51 (75.00)	2 (2.94)	5 (7.35)	10 (14.70)
ITS2	68	57 (83.82)	4 (5.88)	7 (10.29)	52 (76.47)	3 (4.41)	4 (5.88)	9 (13.23)
*mat*K	65	44 (67.69)	16 (24.61)	5 (7.69)	44 (67.69)	15 (23.07)	5 (7.69)	1 (1.53)
*rbc*L	34	7 (20.58)	24 (70.58)	3 (8.82)	7 (20.58)	24 (70.58)	3 (8.82)	0 (0.00)
*trn*H-*psb*A	56	38 (67.85)	6 (10.71)	12 (21.42)	38 (67.85)	6 (10.71)	12 (21.42)	0 (0.00)

Correct: identified; ambiguous; incorrect: unidentified; no match: under threshold. Above number: numbers of sequences; below number: percentage of sequences out of total sequences.

## Appendix A

**Table A1:** List: code, and location collection of the sample vouchers.

	Scientific Name	IUCN 2019	Herbal	Sample Voucher	Collect Location
1	*D. aloifolium* (Bl.) Rchb. f.	LC		18TT	the collection of Biotechnology Center Ho Chi Minh
				18DT	the collection in Duc Trong District, Lam Dong Province, Vietnam
				18PN	the collection in Phu Nhuan District, Ho Chi Minh City, Vietnam
2	*D. amabile* (Lour.) O’ Brien			1DT	the collection in Duc Trong District, Lam Dong Province, Vietnam
				1DT2	the collection in Duc Trong District, Lam Dong Province, Vietnam
				1PN	the collection in Phu Nhuan District, Ho Chi Minh City, Vietnam
3a	*D. anosmum* Lindl.			27TT	the collection of Biotechnology Center Ho Chi Minh
				27DT	the collection in Duc Trong District, Lam Dong Province, Vietnam
				6TT	the collection of Biotechnology Center Ho Chi Minh
				6DT	the collection in Duc Trong District, Lam Dong Province, Vietnam
3b				15TT	the collection of Biotechnology Center Ho Chi Minh
				15DT	the collection in Duc Trong District, Lam Dong Province, Vietnam
				15PN	the collection in Phu Nhuan District, Ho Chi Minh City, Vietnam
4	*D. aphyllum* (Roxb.) C. Fisch. 1928	LC	X	6PN	the collection in Phu Nhuan District, Ho Chi Minh City, Vietnam
5	*D. capillipes* Rchb.f.		X	28DT	the collection in Duc Trong District, Lam Dong Province, Vietnam

				28PN	the collection in Phu Nhuan District, Ho Chi Minh City, Vietnam
6	*D. chrysotoxum Rchb.f;*		X	13TT	the collection of Biotechnology Center Ho Chi Minh
				13DT2	the collection in Duc Trong District, Lam Dong Province, Vietnam
				13PN	the collection in Phu Nhuan District, Ho Chi Minh City, Vietnam
7	*D. cretaceum Lindl. 1847.*			37TT	the collection of Biotechnology Center Ho Chi Minh
				37DT	the collection in Duc Trong District, Lam Dong Province, Vietnam
				37PN	the collection in Phu Nhuan District, Ho Chi Minh City, Vietnam
8	*D. crumenatum Sw.*			34TT	the collection of Biotechnology Center Ho Chi Minh
				34DT	the collection in Duc Trong District, Lam Dong Province, Vietnam
9	*D. crystallinum Rchb. f. (1868)*		X	35TT	the collection of Biotechnology Center Ho Chi Minh
				35DT	the collection in Duc Trong District, Lam Dong Province, Vietnam
				35PN	the collection in Phu Nhuan District, Ho Chi Minh City, Vietnam
10	*D. densiflorum Wall. ex Lindl*			11TT	the collection of Biotechnology Center Ho Chi Minh
				11DT	the collection in Duc Trong District, Lam Dong Province, Vietnam
				11DT2	the collection in Duc Trong District, Lam Dong Province, Vietnam
11	*D. devonianum Paxton (1840)*		X	20TT	the collection of Biotechnology Center Ho Chi Minh
				20DT	the collection in Duc Trong District, Lam Dong Province, Vietnam
				20DT2	the collection in Duc Trong District, Lam Dong Province, Vietnam
12	*D. farmeri Paxton Lindl.f.Rchb.f.*			14DT	the collection in Duc Trong District, Lam Dong Province, Vietnam
				14DT2	the collection in Duc Trong District, Lam Dong Province, Vietnam
				14PN	the collection in Phu Nhuan District, Ho Chi Minh City, Vietnam
13	*D. fimbriatum Hook (1823)*		X	22TT	the collection of Biotechnology Center Ho Chi Minh
				22DT	the collection in Duc Trong District, Lam Dong Province, Vietnam
				22DT2	the collection in Duc Trong District, Lam Dong Province, Vietnam
14	*D. hercoglossum Rchb. f. 1886*		X	21TT	the collection of Biotechnology Center Ho Chi Minh
				21DT	the collection in Duc Trong District, Lam Dong Province, Vietnam
				21PN	the collection in Phu Nhuan District, Ho Chi Minh City, Vietnam
15	*D. intricatum Gagnep (1930)*			36TT	the collection of Biotechnology Center Ho Chi Minh
				36DT	the collection in Duc Trong District, Lam Dong Province, Vietnam
16	*D. linguella Rchb. f. 1882*			33TT	the collection of Biotechnology Center Ho Chi Minh
17	*D. nobile Lindl.*		X	30TT	the collection of Biotechnology Center Ho Chi Minh
				30DT	the collection in Duc Trong District, Lam Dong Province, Vietnam
				30PN	the collection in Phu Nhuan District, Ho Chi Minh City, Vietnam
18	*D. parishii Rchb. f 1863*			38R-DT	the collection in Duc Trong District, Lam Dong Province, Vietnam
19	*D. primulinum Lindl*		X	28TT	the collection of Biotechnology Center Ho Chi Minh
				12TT	the collection of Biotechnology Center Ho Chi Minh
				12DT	the collection in Duc Trong District, Lam Dong Province, Vietnam
				12PN	the collection in Phu Nhuan District, Ho Chi Minh City, Vietnam
20	*D. pulchellum Roxb. ex Lindl.*			10TT	the collection of Biotechnology Center Ho Chi Minh
				10DT	the collection in Duc Trong District, Lam Dong Province, Vietnam
				10DT2	the collection in Duc Trong District, Lam Dong Province, Vietnam
				10PN	the collection in Phu Nhuan District, Ho Chi Minh City, Vietnam
21	*D. salaccense (Bl.) Lindl.*		X	24DT	the collection in Duc Trong District, Lam Dong Province, Vietnam
22	*D. secundum (Bl.) Lindl.*			17TT	the collection of Biotechnology Center Ho Chi Minh
				17DT	the collection in Duc Trong District, Lam Dong Province, Vietnam
23	*D. signatum Rchb. f. 1884*			2DT	the collection in Duc Trong District, Lam Dong Province, Vietnam
				2PN	the collection in Phu Nhuan District, Ho Chi Minh City, Vietnam
				2TT	the collection of Biotechnology Center Ho Chi Minh
24	*D. sulcatum Lindl. (1838)*			5DT	the collection in Duc Trong District, Lam Dong Province, Vietnam
25	*D. superbum Rchb.f.*			3TT	the collection of Biotechnology Center Ho Chi Minh
				3DT	the collection in Duc Trong District, Lam Dong Province, Vietnam
26	*D. tortile Lindl*			32TT	the collection of Biotechnology Center Ho Chi Minh
27	*D. venustum Teijsm. & Binn. 1864*			26TT	the collection of Biotechnology Center Ho Chi Minh
				26DT	the collection in Duc Trong District, Lam Dong Province, Vietnam
				26L	the collection in Long An Province, Vietnam
				29TT	the collection of Biotechnology Center Ho Chi Minh
28	*D. anosmum* × *D. parishi*			38TT	the collection of Biotechnology Center Ho Chi Minh
				38DT	the collection in Duc Trong District, Lam Dong Province, Vietnam
				38DT2	the collection in Duc Trong District, Lam Dong Province, Vietnam
29	*D. anosmum* × *D. aphyllum*			CN	the collection in Duc Trong District, Lam Dong Province, Vietnam
30	*D. Gatton Sunray*			31TT	the collection of Biotechnology Center Ho Chi Minh

## Appendix B

**Table A2:** List of accession numbers of sequences obtained by this study and from Genbank for phylogenetic analysis.

		ITS		matK		rbcL		trnH-psbA	
SPECIES	VOUCHER	Obtained from This Study	Obtained from Genbank	Obtained from This Study	Obtained from Genbank	Obtained from This Study	Obtained from Genbank	Obtained from This Study	Obtained from Genbank
*D. aloifolium*	18DT	MT004837	AY239951.1	MT019381	AB847694.1	Not available	KC660972.1	Not available	
	18TT	MT004836		MT019380		MT019343		MT019476	
	18PN	MT004838		MT019379		Not available		Not available	
*D. anosmum*	3TT	MT004839	JN388570.1	MT019385	KY966807.1	MT019346	KJ944591.1	MT019457	
	3DT	MT004840	KP743544.1	MT019386	AB972311.1	Not available		MT019456	
	15DT	MT004841	MK522219.1	MT019387	MG490279.1	Not available		MT019474	
	15TT	MT004842	KJ944630.1	Not available		MT019345		MT019472	
	15PN	MT004843	AB593499.1	MT019388		Not available		MT019473	
	27TT	MT004844		MT019389		MT019344		MT019489	
	27DT	MT004845		MT019390		Not available		MT019490	
	6TT	MT004846		MT019391		MT019347		MT019459	
	6DT	MT004847		MT019392				MT019460	
*D. aphyllum*	6PN	MT004848	KJ210415.1	MT019393	AB847736.1	Not available		MT019461	
			KJ210414.1		GU565188.1				
			KJ210413.1		KF143640.1				
			KF143430.1						
			HM054561.1						
*D. capillipes*	28DT	MT004849	KY966519.1	MT019395	KF143643.1	Not available	FJ216545.1	MT019493	MF437027.1
	28PN	MT004850	AF362035.1	MT019396	MG490258.1	Not available	KF177576.1	MT019492	
			KF143433.1		MG490256.1				
			HQ114224.1		MF409028.1				
			MK522242.1						
			AB593515.1						
*D. chrysotoxum*	13PN	MT004851	HQ114223.1	MT019398	KF143654.1	Not available	FJ216582.1	Not available	MF437024.1
	13TT	MT004852	HQ114222.1	MT019397	FJ794062.1	MT019349	FJ216544.1	MT019468	MF437025.1
	13DT2	MT004853	HQ114221.1	MT019399	MG490221.1	Not available	FJ216576.1	MT019469	
			HM590383.1		MG490220.1		HM055094.1		
			MK522232.1		KY966816.1		JF713157.1		
			MK483291.1				KT778725.1		
			MK483283.1				HM055093.1		
			MK483272.1						
			MK483266.1						
			KX440955.1						
			KX440953.1						
			AB593533.1						
*D. cretaceum*	37TT	MT004854	KJ944626.1	MT019400	KF957845.1	MT019359	KJ944587.1	MT019512	
	37DT	MT004855	KY966528.1	MT019401	KY966818.1	Not available		MT019510	
	37PN	MT004856		MT019402		Not available		MT019511	
*D. crumenatum*	34TT	MT004864	AY239963.1	MT019403	AB847734.1	MT019350	JF713166.1	MT019500	
	34PN	MT004865	AY273708.1	MT019404	AB972308.1		JF713165.1	MT019501	
			JN388587.1				JF713164.1		
			HM590370.1						
			MK522246.1						
			AB972336.1						
			MH763846.1						
			AB593537.1						
*D. primulinum*	12TT	MT004857	HM054747.1	MT019427	AB847845.1	MT019368	KF177640.1	MT019466	
	12DT	MT004858	HQ114242.1	MT019428	GU565190.1	Not available	FJ216563.1	Not available	
	12PN	MT004859	MK522184.1	MT019429	KF143708.1	Not available	JF713206.1	MT019467	
	28TT	MT004860	KP265001.1	MT019394	FJ794064.1	MT019348	JF713205.1	MT019491	
			MK483269.1		KF957844.1		JF713204.1		
			KT778755.1		AF445450.1		HM055143.1		
			KJ944625.1		MK603116.1		HM055142.1		
			AB593641.1		MG490265.1		HM055141.1		
					MG490264.1		HM055140.1		
					MG490242.1		HM055139.1		
							KT778724.1		
							KJ944586.1		
*D. devonianum*	20TT	MT004861	KJ210443.1	MT019411	AB847744.1	MT019377	KJ187367.1	MT019477	
	20DT	MT004862	KJ210441.1	MT019412	MG490252.1	Not available	FJ216566.1	MT019478	
	20DT2	MT004863	KF143453.1	MT019413		Not available	KJ187368.1	Not available	
			KP743545.1				JF713174.1		
			KC205194.1				JF713173.1		
			HQ114244.1				JF713172.1		
			KT778760.1				KJ944584.1		
			AB593548.1						
*D. fimbriatum*	22DT	MT004869	JN388588.1	MT019418	AB519776.1	Not available	AB519784.1	MT019484	KT792701.1
	22TT	MT004870	KF143461.1	MT019417	AB847758.1	MT019356	KF177603.1	MT019482	
	22DT2	MT004871	HM054637.1	MT019419	GU565189.1	Not available	FJ216550.1	MT019483	
			HM054636.1		KF143671.1		JF713178.1		
			HM054632.1		AF448863.1		JF713177.1		
			HQ114229.1		MK616656.1		HM055105.1		
			HM590392.1		MG490240.1		HM055104.1		
			MK522230.1				HM055103.1		
			MK483290.1				HM055102.1		
			MK483275.1				HM055101.1		
			MK483271.1				KT778732.1		
*D. hercoglossum*	33TT	MT004874	KJ210457.1	MT019423	AB847777.1	MT019362	KJ187382.1	MT019499	
	21TT	MT004909	KF143472.1	MT019420	KF143682.1	MT019366		MT019479	
	21PN	MT004910	KF143471.1	MT019422	KF143681.1	Not available		MT019480	
	21DT	MT004911	KC205188.1	MT019421	KP159292.1	Not available		MT019481	
			HM590381.1		AB972305.1				
			MK522187.1		MG490274.1				
			KP265004.1						
			AB593580.1						
*D. intricatum*	36TT	MT004872	AB593586.1	MT019446		MT019360		MT019504	
	36DT	MT004873		MT019447		Not available		Not available	
*D. nobile*	30TT	MT004875	JN388579.1	MT019424	AB847821.1	MT019363	EF590519.1	MT019495	KT792690.1
	30DT	MT004876	MH120176.1	MT019425	KP159296.1	Not available	AB519785.1	MT019497	
	30PN	MT004877	MH120175.1	MT019426	KY966854.1	Not available	KF177635.1	MT019496	
			MH120174.1				KF177634.1		
			MH120173.1				MK159250.1		
			MH120172.1				MK159249.1		
			MH120171.1				FJ216583.1		
			HM054717.1				FJ216577.1		
			MK522225.1				FJ216570.1		
			HM590382.1				GQ248590.1		
							HM055130.1		
							HM055129.1		
							HM055128.1		
							HM055127.1		
							KT778720.1		
*D. amabile*	1DT	MT004878	MK522209.1	MT019382	AB847690.1	MT019376		MT019451	MF437029.1
	1DT2	MT004879	AB593495.1	MT019384		MT019375		Not available	
	1PN	MT004880		MT019383		Not available		MT019452	
*D. farmeri*	14DT	MT004881	KX600516.1	MT019414	AB847757.1	Not available	HM055100.1	MT019471	MF437022.1
	14PN	MT004882	KJ672671.1	MT019415	KY966830.1	Not available	HM055099.1	MT019470	
	14DT2	MT004883	HM054631.1	MT019416	MF409019.1	MT019355	HM055098.1	Not available	
			HM054630.1						
			KY966540.1						
			AB593561.1						
*D. densiflorum*	11DT2	MT004884	KJ210438.1	MT019410	AB847742.1	Not available	MG025946.1	Not available	MF579382.1
	11TT	MT004885	KJ210436.1	MT019408	KF143661.1	MT019354	FJ216580.1	MT019464	KT792697.1
	11DT	MT004886	KJ210435.1	MT019409	MG490231.1	Not available	JF713171.1	MT019465	
			HQ114255.1		KY966823.1		JF713170.1		
			HQ114254.1		MF409022.1		JF713169.1		
			MK522257.1				JF713168.1		
							JF713167.1		
							HM055096.1		
							KT778728.1		
*D. pulchellum*	10TT	MT004887	KY966577.1	Not available	AB519778.1	Not available	KF177644.1	Not available	
	10DT	MT004888	KJ210492.1	MT019430	AB519777.1	MT019369	AB519789.1	MT019463	
	10DT2	MT004889	KF143503.1	MT019432	KF143712.1	MT019370	AB519790.1	Not available	
	10PN	MT004890	AB593643.1	MT019431	KY966867.1	MT019371		MT019462	
*D. salaccense*			JN388577.1		AF445451.1		KF177648.1		
			KJ210494.1				KF177647.1		
			KF143506.1						
			HQ114260.1						
			MK522259.1						
			KJ210493.1						
*D. secundum*	17DT	MT004892	AY239993.1	MT019435	AB847862.1	Not available		Not available	
	17TT	MT004893	MK522237.1	MT019434	KY966870.1	MT019367		MT019475	
			AB972355.1		AB972327.1				
			AB593660.1						
*D. signatum*	2TT	MT004894	AB972330.1	MT019436	AB972302.1	MT019374	MG324300.1	MT019453	
	2DT	MT004895	AB593662.1	MT019437	AB847864.1	MT019373		MT019454	
	2PN	MT004896		MT019438		MT019372		MT019455	
*D. tortile*	32TT	MT004897	MK522211.1	MT019445	AB847878.1	MT019361		MT019498	
			KY966585.1		KY966874.1				
			EU477511.1						
			AB593678.1						
*D. venustum*	26TT	MT004898	AB847676.1	MT019440	AB847886.1	MT019365		MT019486	
	26DT	MT004899		MT019441		Not available		MT019487	
	26L	MT004900		MT019442		Not available		MT019488	
	29TT	MT004901		MT019443		MT019364		MT019494	
*D. parishii*	38RDT	MT004902	KC568303.1	Not available		Not available		MT019508	
			EU121417.1						
			KY966570.1						
			KX522639.1						
			KC205202.1						
			HM054736.1						
			HM054735.1						
			HM590378.1						
			KJ944629.1						
			MK522227.1						
			MK483284.1						
			AB972344.1						
			AB593630.1						
*D. sulcatum*	5DT	MT004903	KF143517.1	MT019439	KF143726.1	MT019358	KF177658.1	MT019458	MF579383.1
			MK522262.1		KY966873.1		KY440172.1		
			EU477510.1						
			AB593670.1						
*D. hancockii*	24DT	MT004891	JN388591.1	MT019433	AB847771.1	MT019357		MT019485	
			DQ058787.1		GU565195.1				
			AF362025.1		KF143677.1				
			KF143467.1		FJ794051.1				
			HQ114259.1						
			KP159297.1						
			AB593575.1						
*D. crystallinum*	35TT	MT004866	AB593538.1	MT019405	AB847735.1	MT019351	FJ216564.1	Not available	
	35DT	MT004867	KC205205.1	MT019406	GU565192.1	MT019352	KF177590.1	MT019503	
	35PN	MT004868	HQ114243.1	MT019407	KF143657.1	MT019353	KJ944594.1	MT019502	
			KJ944633.1		KF957852.1		KT778733.1		
			KT778764.1		MG490248.1				
					KY966693.1				
					AF445447.1				
*D. Gatton sunray*	31TT	MT004904		MT019444					
*D. anosmum* × *D. parishii*	38TT	MT004905		MT019448		MT019378		MT019505	
	38DT	MT004906		MT019449				MT019507	
	38DT2	MT004907		MT019450				MT019506	
*D. anosmum* × *D. aphyllum*	CN	MT004908						MT019509	

## Appendix C

**Table A3:** Species resolution results based on phylogenetic trees and nucleotide polymorphism.

No	Species	ITS		ITS2		matK		rbcL		trnH-psbA	
		Tree-Based	Indel-Based	Tree-Based	Indel-Based	Tree-Based	Indel-Based	Tree-Based	Indel-Based	Tree-Based	Indel-Based
1	*D. aloifolium*	+		+		+		+		+	
2	*D. amabile*	+		+		+		―		―	
3	*D. anosmum* (synonym name *D. superbum*)	―		―		―		―		―	
4	*D. aphyllum*	+		+		+		―		―	
5	*D. capillipes*	+		+		+		―		―	
6	*D. chrysotoxum*	+		+		+		―		―	
7	*D. cretaceum*	―	+	―		―		―		―	
8	*D. crumenatum*	+		+		+		+		+	
9	*D. crystallinum*	+		+		+		―		―	
10	*D. densiflorum*	+		+		―		―		―	
11	*D. farmeri*	+		+		―		―		―	
12	*D. fimbriatum*	+		+		―		―		―	
13	*D. hercoglossum* (synonym name *D. linguella*)	―		―		―		―		―	
14	*D. intricatum*	+		+		+		―		―	
15	*D. nobile*	―		―		―		―		―	
16	*D. parishii*	+		+		not available		not available		―	
17	*D. primulinum*	―	+	―		―		―		―	
18	*D. pulchellum*	+		+		+		―		+	
19	D. hancockii (previously named *D. salaccense*)	+		+		+		+		+	
20	*D. secundum*	+		+		―		+		―	
21	*D. signatum*	―		―		―		―		―	
22	*D. sulcatum*	+		+		+		―		―	
23	*D. tortile*	―		―		―		―		―	
24	*D. venustum*	+		+		+		+		+	
	**Identified species**	17/24	2	17/24	0	12/23	0	5/23	0	5/24	0
		19/24		17/24							

## Appendix D

**Table A4:** Sequence identification results based on best match/best close match methods.

Species	Voucher	ITS							ITS2							matK							rbcL							trnH-psbA						
		Best Match (%)			Best Close Match (%)				Best Match (%)			Best Close Match (%)				Best Match (%)			Best Close Match (%)				Best Match (%)			Best Close Match (%)				Best Match (%)			Best Close Match (%)			
		Correct	Ambiguous	Incorrect	Correct	Ambiguous	Incorrect	No Match	Correct	Ambiguous	Incorrect	Correct	Ambiguous	Incorrect	No Match	Correct	Ambiguous	Incorrect	Correct	Ambiguous	Incorrect	No Match	Correct	Ambiguous	Incorrect	Correct	Ambiguous	Incorrect	No Match	Correct	Ambiguous	Incorrect	Correct	Ambiguous	Incorrect	No Match
*D. aloifolium*	18TT	X						X	X						X	X			X						X			X				X			X	
	18DT	X						X	X						X	X			X																	
	18PN	X						X	X						X	X			X																	
*D. amabile*	1DT	X			X				X			X				X			X					X			X			X			X			
	1DT2	X			X				X			X				X			X					X			X									
	1PN	X			X				X			X				X			X											X			X			
*D. anosmum*	27TT	X			X				X			X				X			X					X			X			X			X			
	27DT	X			X				X			X				X			X													X			X	
	6TT			X	X				X			X				X			X					X			X			X			X			
	6DT			X				X	X			X				X			X											X			X			
	15TT	X			X				X			X												X			X			X			X			
	15DT	X			X				X			X				X			X											X			X			
	15PN	X			X				X			X				X			X											X			X			
*D. superbum*	3TT	X			X				X			X				X			X					X			X			X			X			
	3DT	X			X				X			X				X			X											X			X			
*D. aphyllum*	6PN			x				x			X				X			X			X										X			X		
*D. capillipes*	28DT	X			X				X			X				X			X											X			X			
	28PN	X			X				X			X				X			X											X			X			
*D. chrysotoxum*	13TT	X						X	X						X	X			X					X			X			X			X			
	13DT2	X			X				X			X				X			X											X			X			
	13PN	X			X				X			X					X			X																
*D. cretaceum*	37TT	X			X				X			X				X			X					X			X			X			X			
	37DT	X			X				X			X				X			X											X			X			
	37PN	X			X				X			X				X			X											X			X			
*D. crumenatum*	34TT	X			X				X			X				X			X					X			X			X			X			
	34PN	X			X				X			X				X			X											X			X			
*D. crystallinum*	35TT	X			X				X			X				X			X				X			X										
	35DT	X			X				X			X				X			X				X			X				X			X			
	35PN	X			X				X			X				X			X				X			X				X			X			
*D. densiflorum*	11TT			X				X	X			X					X			X					X			X				X			X	
	11DT	X			X				X			X					X			X										X			X			
	11DT2	X			X				X			X						X			X															
*D. farmeri*	14DT	X			X				X			X					X			X										X			X			
	14DT2	X			X				X			X					X			X				X			X									
	14PN	X			X				X			X					X			X										X			X			
*D. fimbriatum*	22TT			X				X		X					X	X			X					X			X				X			X		
	22DT	X			X				X			X				X			X											X			X			
	22DT2	X			X				X			X				X			X											X			X			
*D. hercoglossum*	21TT	X			X				X			X				X			X					X			X			X			X			
	21DT	X			X				X						X		X			X										X			X			
	21PN	X						X	X			X				X			X											X			X			
*D. linguella*	33TT			X			X				X			X		X			X					X			X					X			X	
*D. intricatum*	36TT	X			X				X			X				X			X					X			X				X			X		
	36DT	X			X				X			X				X			X																	
*D. nobile*	30TT	X			X				X			X					X			X				X			X			X			X			
	30DT	X			X				X			X					X			X										X			X			
	30PN			X			X				X			X			X			X										X			X			
*D. parishii*	38R-DT			X			X				X			X																		X			X	
*D. primulinum*	28TT	X			X				X			X				X			X				X			X						X			X	
	12TT	X			X				X			X					X			X			X			X				X			X			
	12DT	X			X				X			X				X			X																	
	12PN	X			X				X			X				X			X												X			X		
*D. pulchellum*	10TT	X			X				X			X																								
	10DT	X			X				X			X				X			X					X			X			X			X			
	10DT2	X			X				X			X				X			X					X			X			X			X			
	10PN	X			X				X			X				X			X					X			X			X			X			
*D. salaccense*	24DT			X				X			X				X		X			X				X			X					X			X	
*D. secundum*	17TT	X			X				X			X					X					X			X			X			X			X		
	17DT	X			X				X			X						X			X															
*D. signatum*	2DT		X			X				X			X				X			X				X			X			X			X			
	2PN			X			X			X			X				X			X				X			X			X			X			
	2TT		X			X				X			X					X			X			X			X					X			X	
*D. sulcatum*	5DT	X			X						X				X		X			X				X			X				X			X		
*D. tortile*	32TT			X			X				X			X				X			X			X			X					X			X	
*D. venustum*	26TT	X			X				X			X				X			X				X			X				X			X			
	26DT	X			X				X			X				X			X													X			X	
	26L	X			X				X			X				X			X													X			X	
	29TT	X			X				X			X				X			X				X			X						X			X	

## Appendix E

**Table A5:** Comparison of identification species between our study and the study of Tran et al. (2018) [25].

No	Species	Identified Species Uisng ITS	
		Our Study	Tran et al. (2018)
1	*D. aloifolium*	+	not included
2	*D. amabile*	+	―
3	*D. anosmum* (synonym name *D. superbum*)	―	+
4	*D. aphyllum*	+	+
5	*D. capillipes*	+	+
6	*D. chrysotoxum*	+	+
7	*D. cretaceum*	+	not included
8	*D. crumenatum*	+	not included
9	*D. crystallinum*	+	not included
10	*D. densiflorum*	+	not included
	*D.devonianum*	―	not included
11	*D. farmeri*	+	―
12	*D. fimbriatum*	+	+
13	*D. hercoglossum* (synonym name *D. linguella*)	―	not included
14	*D. intricatum*	+	not included
15	*D. nobile*	―	+
16	*D. parishii*	+	+
17	*D. primulinum*	+	+
18	*D. pulchellum*	+	not included
19	*D. hancockii* (previously named *D. salaccense*)	+	+
20	*D. secundum*	+	not included
21	*D. signatum*	―	not included
22	*D. sulcatum*	+	not included
23	*D. tortile*	―	―
24	*D. venustum*	+	not included
25	*D. anosmum* × *D. parishi*	―	not included
26	*D. anosmum* × *D. aphyllum*	―	not included
27	*D. Gatton Sunray*	―	not included
28	*D. findlayanum*	not included	+
29	*D. moschatum*	not included	+
30	*D. chrysanthum*	not included	+
31	*D. thyrsiflorum*	not included	+
32	*D. wattii*	not included	+
33	*D. jenkinsii*	not included	+
34	*D. haveyanum*	not included	―
35	*D. aduncum*	not included	+
36	*D.brymerianum*	not included	+
37	*D. draconis*	not included	+
38	*D. christyanum*	not included	+
		28 species	23 species
		19 identified species	19 identified species
